# Case Report: Genetic evolution of *Burkholderia pseudomallei* during treatment leading to antibiotic resistance and disease relapse.

**DOI:** 10.12688/wellcomeopenres.24138.2

**Published:** 2025-07-30

**Authors:** Terry John Evans, Chantisa Keeratipusana, Anousone Douangnouvong, Vilayouth Phimolsarnnousith, Davanh Sengdatka, Ko Chang, Koukeo Phommasone, Claire Chewapreecha, Elizabeth A. Ashley, Elizabeth M. Batty

**Affiliations:** 1 Lao-Oxford-Mahosot Hospital-Wellcome Trust Research Unit, Mahosot Hospital, Vientiane, Lao People's Democratic Republic; 2 Faculty of Tropical Medicine, Mahidol Oxford Tropical Medicine Research Unit, Mahidol University, Bangkok, Thailand; 3 Microbiology Laboratory, Mahosot Hospital, Vientiane, Lao People's Democratic Republic; 4 Infectious Diseases Department, Mahosot Hospital, Vientiane, Lao People's Democratic Republic; 5 Department of Clinical Tropical Medicine, Mahidol University, Bangkok, Thailand; 6 Parasites and Microbes, Wellcome Sanger Institute, Cambridge, England, UK; 7 Centre for Tropical Medicine and Global Health, University of Oxford Nuffield Department of Medicine, Oxford, England, UK

**Keywords:** Burkholderia pseudomallei, melioidosis, Lao PDR, southeast Asia, sepsis, antimicrobial resistance

## Abstract

**Background:**

Melioidosis is a significant yet neglected cause of sepsis in tropical regions, particularly in southeast Asia, with poor clinical outcomes. It is a growing threat with an expanding global footprint. The causative organism, *Burkholderia pseudomallei*, is intrinsically resistant to most first-line empiric antibiotic regimens, but acquired resistance to recommended antibiotics for this infection is uncommon. Nonetheless, the genetic determinants of resistance in this species remain poorly elucidated.

**Case presentation:**

A 60-year-old farmer presented in septic shock to a hospital in Laos, and *B. pseudomallei* was grown from blood cultures. Following initial antibiotic treatment with meropenem and co-trimoxazole, his infection relapsed. Several subsequent *B. pseudomallei* isolates from the patient were resistant to multiple antibiotics, and whole genome sequencing demonstrated that this phenotype was associated with a novel 54-kb genomic deletion. This deletion, on chromosome 1, includes the 5’ end of *amrR* – which encodes a regulator of an efflux pump known to be important in conferring meropenem resistance – as well as 46 other genes, some of which have not been characterised. Treatment was targeted to the new antibiogram, requiring a further prolonged intravenous course and second-line oral eradication therapy. The patient made a full recovery.

**Conclusions:**

Mutations in *Burkholderia pseudomallei* lead to increased virulence and drug resistance. Repeat microbiological sampling of patients who do not make clinical improvement as anticipated is essential, with repeat full antimicrobial susceptibility testing on subsequent isolates. Characterisation of drug-resistant mutants is required to understand mechanisms of resistance and to predict phenotypes from whole genome sequencing.

## Plain language summary

Melioidosis is a bacterial infection that is common in tropical and sub-tropical countries. It usually causes pneumonia, but can spread through the bloodstream to various organs. Infections often have very poor outcomes, partly because few antibiotics can be used to treat these infections. In this case, treatment failed likely because a mutation in the genome of the bacteria caused resistance to antibiotics. Compared to other bacteria, little is known about drug resistance in melioidosis, so studying resistant strains by whole genome sequencing provides important information. Deletion of a large part of the genome, including a regulator that controls the number of efflux pumps that move antibiotics out of the cell, was the cause of resistance in this case.

## Introduction

Melioidosis is a devastating disease, caused by infection with the Gram-negative, oxidase-positive bacterium *Burkholderia pseudomallei* ^1^. It is typically thought of as being confined to tropical regions of southeast Asia and northern Australia, where it is a common cause of community-acquired pneumonia, visceral abscesses and bloodstream infection in populations with exposure to soil and water. Accumulating data demonstrate that *B. pseudomallei* has a much larger geographic reach than previously thought, and has now been identified in southeast Asia, the Indian subcontinent, Africa and South America ^2^, and recently it has been declared endemic in the Gulf of Mexico, USA ( https://stacks.cdc.gov/view/cdc/119672; accessed 14 ^th^ April 2025). Transcontinental import of the bacteria is an established route of infection in non-endemic areas and is a growing concern ^3, 4^.

Clinical outcomes of melioidosis are unacceptably poor, with mortality reaching 50% in the regions most affected ^5, 6^. In low-resource settings, this may be due to a combination of poor awareness, limited access to healthcare, delayed or misdiagnosis, and increasing rates of co-morbidities such as diabetes. In addition, *B. pseudomallei* is intrinsically resistant to antibiotics which are typical first-line empiric recommendations for community-acquired pneumonia and sepsis ^7, 8^ – leading to delayed initiation of appropriate treatment.

Treatment is further complicated by acquired antibiotic-resistance mutations – which are fortunately rare. Resistant isolates are more difficult to treat, and use of second-line agents, for example in the prolonged eradication phase, has been associated with relapse – even in wild-type strains ^9^. The European Committee on Antimicrobial Susceptibility Testing (EUCAST) has provided antibiotic susceptibility breakpoints for only eight antibiotics. Evolution of decreased susceptibility to meropenem may simultaneously be associated with decreased susceptibility to doxycycline, co-trimoxazole, ceftazidime, and co-amoxiclav ^10^. Many sequence-based resistance-prediction tools that are available online fail to identify resistance determinants in *B. pseudomallei –* in large part because many resistance genes catalogued in the databases are present in all *B. pseudomallei* strains, hence explaining intrinsic resistance. More recently, the Antimicrobial Resistance Detection and Prediction (ARDaP) software tool has been developed, which is specifically tailored to *B. pseudomallei* and provides the most reliable predictions ^11^.

Finally, being a disease that predominantly affects low- and middle-income countries, and previously assumed to be of limited global concern, research into optimal diagnosis and treatment has been inadequate. Indeed, some groups consider melioidosis a neglected tropical disease (NTD) ^12^, not least because its disease burden is higher than many recognised NTDs, although it is not officially recognised as such by the World Health Organization ^13^.

This case describes a novel mutation associated with antibiotic resistance in *B. pseudomallei*, and underscores the value of sequencing and bioinformatics capacity in low- and middle-income countries in order to better understand diseases of global concern.

## Methods

### Bacterial culture and antimicrobial susceptibility testing

Samples were processed following local standard protocols. In brief, after initial processing including Gram stain, samples were cultured on blood agar (Oxoid, incubated in 5–10% CO _2_ for 48 hours), Ashdown’s agar (made in-house, incubated in air and read daily for 4 days), and Brilliance UTI agar (Oxoid) for urines and checked daily. Organisms that grew on culture were identified using standard biochemical and API tests (bioMérieux, Marcy L’Etoile, France) and MALDI-ToF (bioMérieux). Comprehensive antimicrobial susceptibility testing (AST) profiles were determined using disc diffusion on Mueller-Hinton agar (Oxoid); for minimum inhibitory concentration (MIC) determination, antibiotic gradient strips (Liofilchem®) were used. Both methods were performed in line with EUCAST methodology, and interpreted according to breakpoint tables version 13.1 ^14^. For repeat AST testing or DNA extraction, strains were re-streaked from frozen stocks, with a further single colony taken for testing.

### Whole genome sequencing and bioinformatics

DNA was extracted from overnight cultures: 10ml Selective Broth (10g tryptone soya broth, 40ml glycerol, and 5ml 0.1% crystal violet in 1000ml deionised water, supplemented with colistimethate final concentration 50mg/l) was inoculated with a single colony of each *B. pseudomallei* isolate and DNA extracted using the GeneJET Genomic DNA Purification kit (Thermo Fisher Scientific) and quantified using Qubit High Sensitivity assay (Invitrogen). Sequencing was performed at MicrobesNG ( www.microbesng.com) on Illumina NovaSeq 6000 using 2 x 250bp paired-end read kits. Coverage was 49-117X across samples after read trimming (Supplementary Table 1 ^15^).

Illumina read quality control was conducted using FastQC v0.12.1 (available at https://www.bioinformatics.babraham.ac.uk/projects/fastqc/). Genomes were assembled using SPAdes ^16^ and annotated with Prokka v1.14.6 ^17^. Multi-locus sequencing typing (MLST) was performed to classify the samples using mlst (available at https://github.com/tseemann/mlst), and information on other isolates with the same sequence type was obtained from the *B. pseudomallei* PubMLST database ^18^. Core genome multi-locus sequence typing (cgMLST) was performed by uploading whole genome assemblies in fasta format to PubMLST ^18^. Variant calling was carried out using VariantDetective v1.0.1 ^19^ using *B. pseudomallei* K96243 (assembly GCF_000011545.1) as the reference genome and requiring SNP calls to be called by 2 out of 3 variant callers. Antimicrobial resistance profiling for *B. pseudomallei* was performed using Antimicrobial Resistance Detection and Prediction (ARDaP) v1.8.2 ^11^ and NCBI AMRFinderPlus ^20^.

### Case presentation

A 60-year-old gardener from Vientiane, Laos, presented to Mahosot Hospital with a 4-day history of fever, dry cough, lethargy, orbital pain and watery stools. His past medical history included hypertension and type 2 diabetes managed with metformin. He was a smoker.

Auscultation revealed crepitations in the left upper zone, and a clinical diagnosis of lobar pneumonia was made. ECG demonstrated atrial fibrillation with a fast ventricular response (159 beats per minute). Given the local epidemiology of febrile illness, infection work-up ruled out dengue, rickettsial infection and tuberculosis.

Due to septic shock, the patient was admitted to the Intensive Care Unit for intubation and ventilation, and inotropic support, requiring noradrenaline on days 1 to 4. Initial temperature was 39.5°C. The patient was treated empirically with ceftriaxone 2g OD IV and ciprofloxacin – although treatment with ceftazidime and amikacin is recommended by national guidelines in this scenario.

A blood culture taken on admission subsequently grew *B. pseudomallei* susceptible to all antibiotics tested (ceftazidime, meropenem, co-trimoxazole, co-amoxiclav, and tetracycline), as determined by EUCAST disc diffusion methodology and breakpoint interpretation ^14, 21^. Antibiotics were therefore switched to meropenem 1g three times daily. Co-trimoxazole 1440mg twice daily was added on day 7, possibly because the patient remained febrile (>38.0°C).

An abdominal ultrasound was performed and excluded hepatic and splenic abscesses, although multiple bilateral renal stones were noted, causing severe dilation of the left ureter.

**Figure 1.**
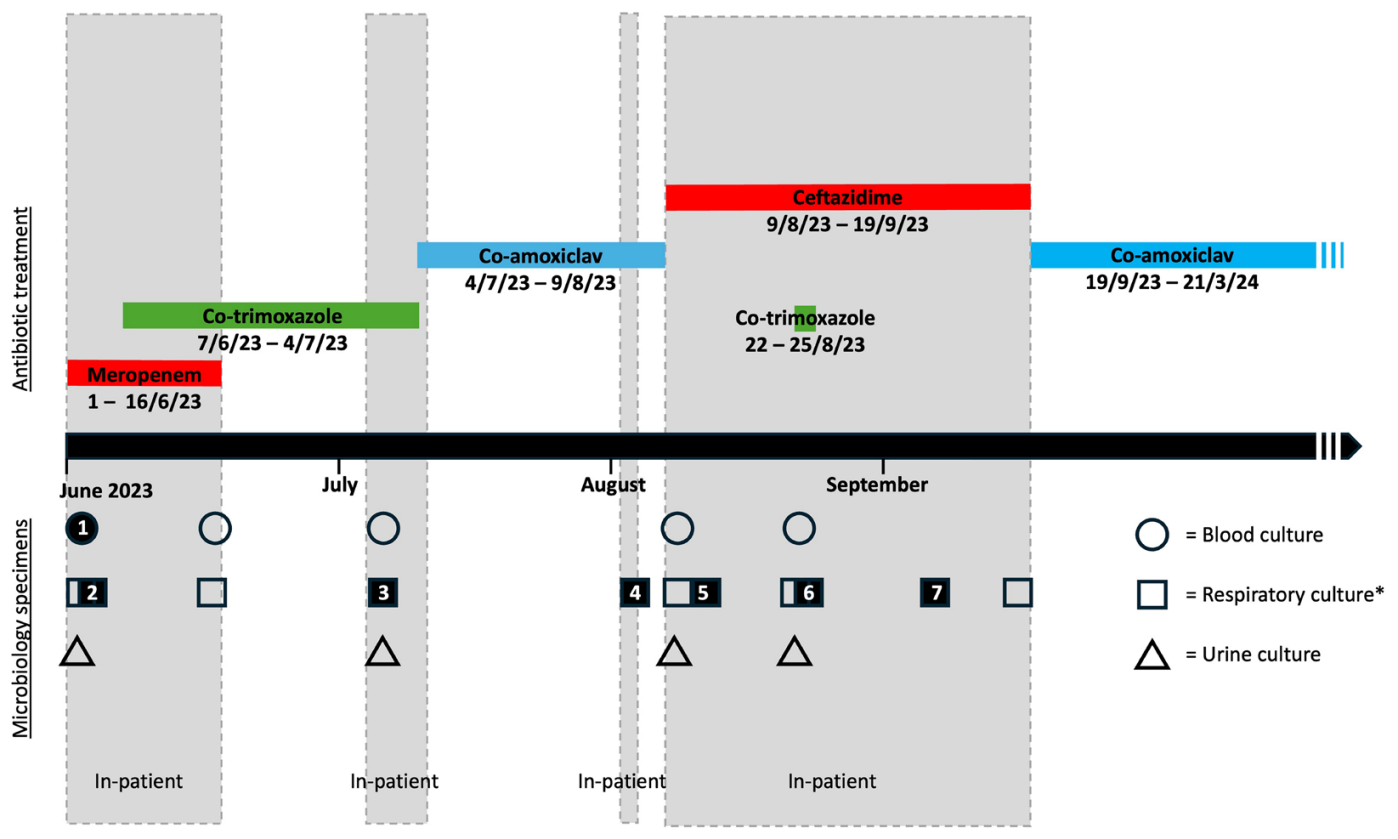
Timeline of cultures and anti- *B. pseudomallei* treatment. All positive cultures are respiratory except the initial blood culture. Solid shapes represent positive cultures; hollow shapes represent negative cultures. *Respiratory cultures include sputum, throat swab, or endotracheal secretions.

**Figure 2.**
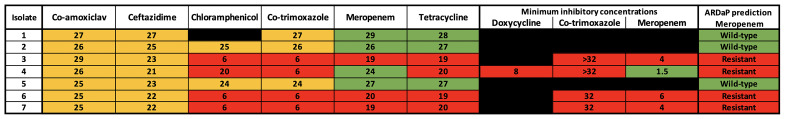
Antimicrobial susceptibility testing of the seven *B. pseudomallei* isolates from this study. Numbers represent inhibition zone diameters (mm). MIC = minimum inhibitory concentration (μg/ml). ARDaP predictions for meropenem are given in the final column. Green shading = susceptible; orange shading = susceptible at increased exposure; red shading = resistant; black = not tested (either due to reagent availability, or because MIC testing was not routine).

The patient was extubated on day 12 and continued to receive co-trimoxazole orally. He was discharged to complete eradication therapy with co-trimoxazole, as per first-line treatment protocols. However, the patient represented about 2 weeks after discharge with ongoing symptoms and a mild neutrophilia of 10.6 x 10 ^3^/μl (reference range: 6.0 – 8.0 x 10 ^3^/μl), although he was afebrile. There was no concern about the patient’s adherence to therapy. Microbiological investigations were repeated. While urine and blood cultures were sterile, *B. pseudomallei* was isolated from a throat swab. This isolate was resistant to meropenem, co-trimoxazole, tetracycline and chloramphenicol. The patient’s antibiotic therapy was switched to co-amoxiclav 1250mg three times daily and he was discharged 4 days later. A third presentation occurred 4 weeks later when the patient described further haemoptysis; he was admitted to hospital for 72 hours; sputum was the only sample taken for microbiological testing on this admission. This was positive for *B. pseudomallei* sensitive to co-amoxiclav, and oral co-amoxiclav therapy was continued. However, following a case discussion with experts in the management of melioidosis, the patient was subsequently recalled to hospital for a 6-week course of intravenous ceftazidime 2g three times a day due to his recalcitrant infection, and because intravenous ceftazidime has been shown to be superior to co-amoxiclav. The patient was then discharged on co-amoxiclav 1250mg three times daily once again to complete a further 24 weeks’ treatment (until 21 ^st^ March 2024). He remains well, with no further positive cultures at 12 months’ follow-up (as of March 2025). A timeline of admissions, culture results and antibiotic treatment is shown in Figure 1 and Table 1. Detailed antimicrobial susceptibility testing results as determined when the bacterial strains were originally isolated are summarised in Figure 2 (results of repeat antimicrobial susceptibility testing at later dates are shown in Supplemental Figure 1).

**Table 1.** Summary of all microbiology samples submitted for this patient.

Admission	Sample number	Sample date	Sample type	Result
1		31/05/2023	Urine	No growth
		01/06/2023	Throat swab	No growth
	1	01/06/2023	Haemoculture	*B. pseudomallei*
	2	02/06/2023	Endotracheal secretions	*B. pseudomallei*
		14/06/2023	Throat swab	No growth
		14/06/2023	Haemoculture	No growth
2		05/07/2023	Haemoculture	No growth
		05/07/2023	Urine	No growth
	3	05/07/2023	Throat swab	*B. pseudomallei*
3	4	03/08/2023	Sputum	*B. pseudomallei*
4		09/08/2023	Throat swab	No growth
		09/08/2023	Urine	No growth
		09/08/2023	Haemoculture	No growth
	5	12/08/2023	Sputum	*B. pseudomallei*
		21/08/2023	Haemoculture	No growth
		21/08/2023	Urine	No growth
		21/08/2023	Throat swab	No growth
	6	22/08/2023	Sputum	*B. pseudomallei*
	7	07/09/2023	Sputum	*B. pseudomallei*
		14/09/2023	Sputum	No growth

Given the unexpected resistance phenotype, antimicrobial susceptibility testing was repeated several times for confirmation. While the results were largely consistent, there was some variability, especially with results that were close to EUCAST breakpoints (Supplemental Figure 1 ^15^). 

The drug resistance phenotypes observed could either be explained by a pre-existing resistant sub-population, in-host mutation and evolution under antibiotic selective pressure, or even due to initial infection with two distinct isolates with differing susceptibility patterns ^22– 24^. To investigate these possibilities further, whole genome sequencing was performed for all seven isolates.

MLST has previously been used to identify clonality in *B. pseudomallei* ^25^ and to determine if a recurrent infection is a relapse, or a reinfection with a different strain present during the initial infection ^6, 9^. All seven of our isolates were sequence type ST-34 and complete genome sequence type cgST-753. ST-34 isolates have previously been reported in Thailand, Cambodia and Malaysia. This suggested that all seven isolates are from a single clone, and this is a relapse of the initial infection.

We searched for potential genotypic determinants of resistance from our whole genome sequencing data using NCBI AMRFinderPlus, which covers multiple bacterial species, and ARDaP, which is designed specifically for *B. pseudomallei.* Using NCBI AMRFinderPlus we identified that the *bla*_OXA-57_ carbapenemase gene was present in all isolates. This gene has previously been identified in *B. pseudomallei* ^26, 27^, and is present in 33% of *B. pseudomallei* isolates based on sequence data uploaded to the NCBI database ( https://card.mcmaster.ca/ontology/38171), but it has not been shown to cause phenotypic resistance to carbapenems ^28^.

ARDaP additionally predicted meropenem monoresistance for four isolates through loss of the *amrR* gene (BPSL1805), which acts as a local regulator of the AmrAB efflux pump. This results in upregulation of the efflux pump, reducing susceptibility to meropenem and potentially conferring full resistance to it. ARDaP gave no prediction for the remaining antibiotics. The remaining three fully sensitive isolates showed no detectable antibiotic resistance markers.

By mapping sequencing reads to *B. pseudomallei* K96243 (GCF_000011545.1), we confirmed a deletion which removes the 3’ end of the *amrR* gene in the isolates with predicted meropenem resistance ( Figure 3A). The deletion of approximately 54 kb on chromosome 1 from 2,152,753 to 2,207,298 bp removes 46 genes (listed in Supplemental Table 2 ^15^) and is immediately upstream of the *amrAB* operon which is regulated by AmrR; the coding regions of *amrA* and *amrB* themselves are not affected by the deletion ( Figure 3B).

**Figure 3A.**
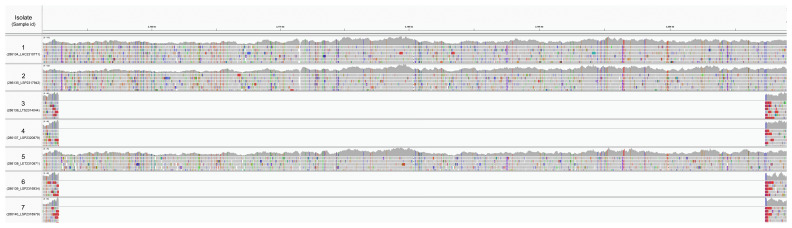
Integrative Genomics Viewer visualization of the seven samples aligned to the *B. pseudomallei* K96243 genome. The four meropenem-resistant samples show a deletion on chromosome 1 between 2,152,753 and 2,207,298 bp.

**Figure 3B.**
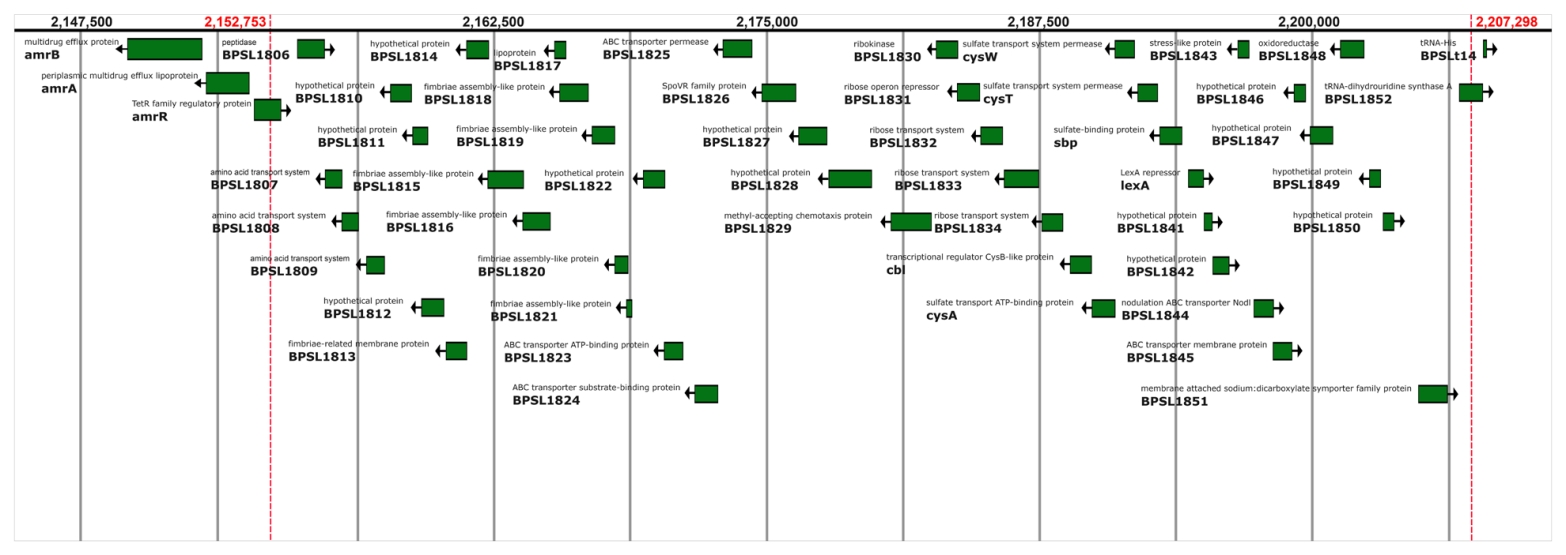
A diagram showing the genes present in the deleted region in the meropenem-resistant isolates. *amrA* and *amrB* are outside the deletion.

Finally, we looked at single nucleotide polymorphisms and small insertions and deletions in the strains using VariantDetective. No additional polymorphisms were found in these seven strains, suggesting that no further genetic determinants of resistance existed in these strains.

## Discussion

Melioidosis remains an important cause of sepsis in many tropical countries including Laos, where it is one of the commonest species isolated from blood cultures. As this case highlights, diabetes is a common risk factor, and patients typically present in a severe condition.

In melioidosis, blood cultures and other clinical specimens may remain positive for longer than is typically seen with other Gram-negative pathogens – likely due to the high bioburden, biofilm production and tendency for abscess formation. This does not necessarily imply treatment failure. Reasons for failure or relapse include non-compliance with treatment or presence of an undrained collection. A CT scan was not performed in this case, and we do not routinely investigate for prostatic abscesses in our setting due to resource limitations, although these were found in 15–21% of Australian men with melioidosis ^29, 30^. Failing to use co-trimoxazole for the eradication phase of treatment also increases the chance of treatment failure ^9, 31^, but this was unavoidable in this case due to co-trimoxazole resistance.

We identified a novel partial deletion of *amrR*. This is a TetR-family transcriptional regulator that regulates expression of the AmrAB-OprA efflux pump. Absence of AmrR causes overexpression of the AmrAB efflux pump and has been previously implicated in meropenem-resistance, including in the development of resistance during treatment ^9, 10, 32, 33^, and is also involved in doxycycline resistance ^34^. A recent study demonstrated that the phenotype of 3 clinical isolates with mutations in *amrR* that were less susceptible to meropenem was reversed when a wild-type copy of the *amrR* gene was provided on a plasmid. RNA expression levels of the *oprA*, *amrA* and *amrB* genes in the *amrR* mutants were re-repressed in the presence of the wild-type *amrR* gene, confirming the role of *amrR* as a regulator of this efflux pump ^35^.

Previous comparisons of paired isolates from initial infections and relapses have demonstrated that a wide range of genetic and genomic alterations occur, including point mutations, and insertions and deletions. One such large-scale deletion involved a 330-kb region encompassing a *tetR* gene ^36^. Presumably these striking genomic changes, which were associated with reduced antibiotic susceptibility, are selected for in the host and confer a survival advantage.

While meropenem resistance is typically mediated by overexpression of the AmrAB-OprA efflux pump, the majority of co-trimoxazole, tetracycline, and chloramphenicol resistance is mediated by overexpression of an alternative efflux pump, BpeEF-OprC. This pump is located on chromosome 2, but is regulated by several LysR regulators spanning chromosomes 1 and 2 ^37^. Of these regulators, only BpeS (chromosome 1) and BpeT (chromosome 2) are well-studied. However, there is an uncharacterised LysR regulator (BPSL1835; *cbl*) in the 54-kb deletion in our isolates that might plausibly affect BpeEF-OprC expression. Apart from *amrR*, there were no mutations in genes identified which have been previously described as a genotypic cause of resistance. We did not investigate the transcript levels of any genes involved in resistance.

Using NCBI AMRFinderPlus, we identified a *bla*_OXA-57 _carbapenemase resistance gene, first identified in *B. pseudomallei* and *B. thailandensis* isolates ^28^. *bla*_OXA_ beta-lactamase genes are common in *B. pseudomallei* strains but have not been implicated in conferring carbapenem resistance ^38^. *In vitro* biochemical data have shown that while the *bla*_OXA-57_ enzyme can hydrolyze penicillins and first-generation cephalosporins, it only slowly hydrolyzes imipenem and meropenem – activity which remains of unproven *in vivo* significance ^39^. If it is shown to contribute to carbapenem resistance, perhaps in combination with other mechanisms, this may support the preferred use of ceftazidime for the treatment of melioidosis because *bla*_OXA-57_ has no hydrolytic activity against ceftazidime at all.

The reasons for variable AST results on repeat testing in our study are unclear. The observation may be due to hetero-resistance – a phenomenon whereby a pre-existing minor sub-population of bacterial cells are antibiotic-resistant, and become dominant following antibiotic exposure. This phenomenon is increasingly recognised as a cause of discrepant AST results, because causative mutations are often unstable or reversible ^40^. Indeed, Schnetterle *et al*. have described a resistance phenotype in a *B. pseudomallei* strain that is unstable on serial passage ^33^. Such dynamic changes in resistance phenotype have been dubbed ‘adaptive resistance,’ and this appears to be particularly important in efflux pump-mediated resistance ^41^, and may be due to epigenetic mechanisms of gene regulation ^42^.

Resistance to meropenem as predicted by ARDaP largely correlated with *in vitro* testing, but was discrepant in a small number of cases – particularly evident for isolate 2. This may reflect a hetero-resistant phenotype, whereby the colony picked for AST testing had a different genetic background to the colony used for genomic DNA extraction and sequencing.

A further limitation of our study is that we sequenced only a single colony from each time point, and this may have caused mutations present in only a sub-population of cells to go undetected. Of note, on the original culture plates used for isolating *B. pseudomallei* strains, we did not observe varying colony morphotypes, and so there were not obvious sub-populations.

We typically use monotherapy for the treatment of melioidosis in our hospital, but the Darwin protocol ^43^ advocates dual therapy (intravenous ceftazidime or meropenem with oral co-trimoxazole) for the intensive phase where there is cutaneous melioidosis, osteomyelitis, septic arthritis, CNS involvement or with deep-seated collections; this is for improved tissue penetration and ‘to potentially limit the emergence of resistance.’ However, there is currently no trial data demonstrating benefit of dual therapy in either the intensive or eradication phase of therapy ^44– 46^. The latest Darwin guidelines also recommend a prolonged intravenous intensive phase in some circumstances: if there is pneumonia with either lymphadenopathy or ITU admission, multilobar unilateral or bilateral pneumonia with positive blood cultures, or deep-seated collection (4 weeks minimum); osteomyelitis (6 weeks minimum); or central nervous system infection or arterial infection (8 weeks minimum). Carefully controlled trials are required to determine the optimal treatment of this devastating infection, although additional co-trimoxazole would likely not have prevented relapse in this case given that it had minimal discernible inhibitory effect on our resistant isolates.

In summary, we present a case study of a patient with persistent symptoms and a suspected melioidosis relapse showing a change in antibiotic susceptibility in sputum culture over time. Later isolates show resistance to meropenem and co-trimoxazole, with the only genetic change being a large deletion involving a known meropenem resistance gene and several other *B. pseudomallei* genes which are not well characterised. Repeat sampling and antimicrobial resistance testing is required when patients do not respond to treatment as anticipated to check for alterations in susceptibility profile and to allow prescription of more appropriate antibiotics. A greater understanding of resistance mechanisms that occur during long-term antibiotic treatment for melioidosis is required, and genomic comparison of serial isolates from a single patient may identify novel mutations and resistance mechanisms.

## Public and patient involvement

There was no formal patient or public involvement in the design or conduct of the study.

## Consent for publication

Written informed consent for publication was obtained from the patient.

## Ethics and consent

Ethical approval was not required. Written informed consent was obtained from the patient for the publication of this case report.

## Data Availability

Whole genome sequence data are uploaded to the Sequence Read Archive under project PRJNA1238472 with URL https://www.ebi.ac.uk/ena/browser/view/PRJNA1238472. **Figshare:** Supplemental data are available at Figshare under project “Genetic evolution of *Burkholderia pseudomallei* during treatment leading to antibiotic resistance and disease relapse – a case report.” https://doi.org/10.6084/m9.figshare.29497496.v1 ^15^. This project contains the following underlying data: Evans *et al.* 2025 BPS case report Supplemental file.doc Data is available under CC BY4.0 license. **Figshare:** Evans *et al.* 2025 CARE checklist.pdf, https://doi.org/10.6084/m9.figshare.28921367.v1 ^15^ Data is available under CC0 license.
