# Supplementary material for: Case Report: Genetic evolution of Burkholderia pseudomallei during treatment leading to antibiotic resistance and disease relapse
Source: Wellcome Open Res. 2025 Jul 30;10:281. Originally published 2025 May 28. [Version 2] doi: 10.12688/wellcomeopenres.24138.2 (PMC12374155; doi:10.12688/wellcomeopenres.24138.2)
Supplement: Peer review history

# Reviewer report: Reviewer response for version 2

**DOI:** 10.21956/wellcomeopenres.27137.r127913

**Published:** 2025-08-22

**Authors:** Fabienne Neulat-Ripoll

**Affiliations:** 1 French Army Biomedical Research Institute, Brétigny-sur-Orge, Île-de-France, France

I have no comments to add to this new version.

Are enough details provided of any physical examination and diagnostic tests, treatment given and outcomes?

Partly

Is the case presented with sufficient detail to be useful for other practitioners?

Partly

Is sufficient discussion included of the importance of the findings and their relevance to future understanding of disease processes, diagnosis or treatment?

Partly

Is the background of the case’s history and progression described in sufficient detail?

Yes

Reviewer Expertise:

Melioidosis, antibiotic resistance mechanisms, with a particular focus on membrane-associated mechanisms such as efflux, as well as genomics and transcriptomics.

I confirm that I have read this submission and believe that I have an appropriate level of expertise to confirm that it is of an acceptable scientific standard.

# Reviewer report: Reviewer response for version 2

**DOI:** 10.21956/wellcomeopenres.27137.r127914

**Published:** 2025-08-12

**Authors:** Nicole L. Podnecky

**Affiliations:** 1 Saint Michael's College, Colchester, USA

I have no further comments to add to this report.

Are enough details provided of any physical examination and diagnostic tests, treatment given and outcomes?

Partly

Is the case presented with sufficient detail to be useful for other practitioners?

Yes

Is sufficient discussion included of the importance of the findings and their relevance to future understanding of disease processes, diagnosis or treatment?

Yes

Is the background of the case’s history and progression described in sufficient detail?

Yes

Reviewer Expertise:

Antimicrobial resistance in Gram negatives (Burkholderia spp., E. coli), Bacteriology, Molecular genetics

I confirm that I have read this submission and believe that I have an appropriate level of expertise to confirm that it is of an acceptable scientific standard.

# Reviewer report: Reviewer response for version 1

**DOI:** 10.21956/wellcomeopenres.26629.r124546

**Published:** 2025-07-03

**Authors:** Nicole L. Podnecky

**Affiliations:** 1 Saint Michael's College, Colchester, USA

In the article titled “Case report: Genetic evolution of *Burkholderia pseudomallei* during treatment leading to antibiotic resistance and disease relapse – a case report” Evans, et al. describe a large genomic deletion that caused altered antimicrobial susceptibilities of clinical specimens.  Since *B. pseudomallei* is intrinsically resistant to many antimicrobials, the development of additional resistance traits can greatly limit the therapeutic options for patient care.  In this case study, a patient initially treated with meropenem required subsequent in-patient care on three occasions and several attempted therapeutics until resolution following ceftazidime followed by co-amoxiclav treatment.  Follow-up analyses including whole genome sequencing elucidated a probable cause of meropenem resistance - a large genomic deletion which interrupted *amrR*, a known transcriptional regulator of the AmrAB efflux pump.  AmrAB overexpression confers resistance to aminoglycosides and macrolides, as well as meropenem. Nimnuan-ngam S, et al 2025 (Ref 1)

 Overall, the report is well-written, detailed, and presents data and findings that are valuable to those interested in better understanding resistance development and strain variation & mutation during persistent infections with *B. pseudomallei*.  The case study provides insight into patient care and evidence supporting the value of subsequent susceptibility testing and genomic analysis.

 While, it would have been ideal to have RT-qPCR or similar analysis results to validate the overexpression of AmrAB as a result of *amrR* truncation and confirm the cause of meropenem resistance (Webb et al, 2017 & Nmnuan-ngam, 2025) (Ref 2 and 1), there is good experimental evidence that demonstrate this transcriptional repressor role of AmrR on *amrB* expression (Webb et al. 2017) (Ref 2)

 // Major Comments

 1.  The antimicrobial susceptibility testing (AST) methods appear incomplete in their current form.  In the **Case Presentation**, it appears that AST differed across the various patient samples, since initially susceptibility testing was described as: “ *EUCAST by EUCAST methodology and breakpoint interpretation 14,21*” then later the methodology is described as “ *by disk diffusion testing*.”  This gives the impression that testing guidelines/breakpoints may have varied since they were clearly outlined initially.  

 Similarly in **Figure 2**, it is unclear why the authors have some antimicrobials (doxycycline, co-trimoxazole, and meropenem) tested by disk diffusion as well as have MICs reported.  MICs cannot be determined by disk diffusion alone, but this was the only methodology described in the methods section.  Please ensure this is clarified.

 I urge the authors to be either consistent in the descriptions of the methods or specifically discuss differences and how this could impact interpretation/breakpoint values. 

 // Minor Comments & suggestions

**Referencing**

 I encourage the authors to include reference to the following relevant articles:  

**Nimnuan-ngam et al. 2025** (Ref 1), a very recent paper which supports a relationship between *amrR* deletion/mutation and meropenem resistance.

 The discussion section could be improved by referencing other relevant examples of large genetic deletions and rearrangements that were described in relapsing infections and often led to changes in antimicrobial susceptibilities:

**Hayden et al, 2012** (Ref 3), describes the longitudinal changes to *B. pseudomallei* isolates comparing initial and relapsing infections.  Most relevantly they describe a 330 Kb deletion that interrupted *tetR*, a 800 bp inversion that interrupted *bpeT*, and tandem repeats including PBP3 which all affected antimicrobial susceptibilities.

 Other examples are more deleterious – showing genome reduction and loss of antimicrobial resistance, which may be beyond the scope of this manuscript, but does illustrate the genomic plasticity of *B. pseudomallei*:

**Price et al, 2013** (Ref 4)

**Trunck et al, 2009** (Ref 5)

**Title:**  case report is mentioned twice in the title and feels redundant this may be due to the emphasis of both the journal and the authors.

**Plain Language Summary:  **Within the PLS there are a few concepts that could be modified to improve comprehension of the writing for a general audience. 

 For example the use of the word “Comparatively” should clearly distinguish what is being compared:  Suggest to revise to something like:  “Compared to other bacteria, little is known…”  

 I also suggest modification of the last sentence to clarify “regulator of an efflux pump”: *Deletion of a large part of the genome, including a [regulator that controls the number of efflux pumps that moves antibiotics out of the cell,] was the cause of resistance in this case.*

**Introduction:**

 Line 7-8:  The sentence “ *In children, localised skin abscesses and parotitis are also common*”. Feels disruptive in between sentences on the discussion of geographic distribution – I recommend the authors slightly revise this section to discuss geography then disease state separately.

**Figure 1**

 Please specify in the figure legend that the shape (symbol) indicates the type of microbiological sample tested.  Where circle = blood, square = respiratory, and presumably triable = urine.

**Discussion**

 Just a general comment - It is interesting the authors found no other SNP mutations between isolates, especially those with deletion of *lexA*, however since there are multiple copies of this gene the regulation of the SOS response is controlled. However, *B. pseudomallei* (as mentioned above) is prone to deletions and rearrangements – as demonstrated in other studies on paired clinical isolates that have developed resistance.

Are enough details provided of any physical examination and diagnostic tests, treatment given and outcomes?

Partly

Is the case presented with sufficient detail to be useful for other practitioners?

Yes

Is sufficient discussion included of the importance of the findings and their relevance to future understanding of disease processes, diagnosis or treatment?

Yes

Is the background of the case’s history and progression described in sufficient detail?

Yes

Reviewer Expertise:

Antimicrobial resistance in Gram negatives (Burkholderia spp., E. coli), Bacteriology, Molecular genetics

I confirm that I have read this submission and believe that I have an appropriate level of expertise to confirm that it is of an acceptable scientific standard, however I have significant reservations, as outlined above.

## References

[1] : Identification of novelamrR deletions as meropenem resistance mechanisms in clinicalBurkholderia pseudomallei isolates. *Microbiology Spectrum* .2025;13(5) : 10.1128/spectrum.01936-24 10.1128/spectrum.01936-24 PMC1205404540135925

[2] : Loss of Methyltransferase Function and Increased Efflux Activity Leads to Doxycycline Resistance in Burkholderia pseudomallei. *Antimicrobial Agents and Chemotherapy* .2017;61(6) : 10.1128/AAC.00268-17 10.1128/AAC.00268-17 PMC544412328348161

[3] : Evolution of Burkholderia pseudomallei in Recurrent Melioidosis. *PLoS ONE* .2012;7(5) : 10.1371/journal.pone.0036507 10.1371/journal.pone.0036507 PMC335290222615773

[4] : Within-Host Evolution of Burkholderia pseudomallei over a Twelve-Year Chronic Carriage Infection. *mBio* .2013;4(4) : 10.1128/mBio.00388-13 10.1128/mBio.00388-13 PMC373512123860767

[5] : Molecular Basis of Rare Aminoglycoside Susceptibility and Pathogenesis of Burkholderia pseudomallei Clinical Isolates from Thailand. *PLoS Neglected Tropical Diseases* .2009;3(9) : 10.1371/journal.pntd.0000519 10.1371/journal.pntd.0000519 PMC273763019771149

# Reviewer report: Reviewer response for version 1

**DOI:** 10.21956/wellcomeopenres.26629.r124547

**Published:** 2025-07-03

**Authors:** Fabienne Neulat-Ripoll

**Affiliations:** 1 French Army Biomedical Research Institute, Brétigny-sur-Orge, Île-de-France, France

The article presents the case of a 60-year-old farmer from Laos with diabetes, who was hospitalized in septic shock. Blood cultures identified *Burkholderia pseudomallei*. After initial empirical treatment with ceftriaxone and ciprofloxacin, the patient was switched to meropenem and co-trimoxazole once the microbiological results became available.

 Despite initial improvement, the patient experienced several relapses:

 · **First relapse:** isolation of resistant *B. pseudomallei* from a throat swab.

 · **Second relapse:** positive sputum cultures for resistant *B. pseudomallei*.

 · **Successive treatments:** co-amoxiclav, followed by a 6-week course of intravenous ceftazidime, and then an extended course of oral antibiotics.

 After 12 months of follow-up, no further positive cultures were found.

 The methods used in this study included:

 ·         microbiological analyses (blood, urine, throat swab and sputum cultures),

 ·         antimicrobial susceptibility testing according to EUCAST recommendations,

 ·         and whole genome sequencing (WGS) on 7 isolates using Illumina technology, with typing by MLST and cgMLST.

 The main results showed that all isolates belonged to sequence type ST-34, confirming a relapse rather than a reinfection. Whole genome sequencing revealed a 54-kb genomic deletion on chromosome 1 in the meropenem-resistant strains, including:

 ·         the loss of the *amrR* gene, a repressor of the *AmrAB-OprA* efflux pump responsible for meropenem resistance,

 ·         and the loss of 46 other genes, some of which remain uncharacterized.

 A variability in antimicrobial susceptibility testing results was observed, likely linked to heteroresistance phenomena. The resistance appears to have developed under antibiotic selective pressure during treatment, although initial infection with multiple strains cannot be excluded.

 This case highlights the diagnostic and therapeutic challenges of melioidosis in resource-limited settings. It emphasizes the importance of:

 ·         repeating cultures and susceptibility testing in cases of treatment failure or clinical relapse,

 ·         and developing local genomic sequencing and bioinformatics capacity.

 Finally, this report underlines the need to better understand resistance mechanisms beyond *amrR* loss, particularly those responsible for co-trimoxazole resistance.

**Comments and Suggestions for the Authors**

 1. **Clarification of MIC determination methods:**

 In the *Materials and Methods* section, the methodology used to determine the minimum inhibitory concentrations (MICs) is not specified. MIC determination typically requires broth microdilution assays or E-test strips with an antibiotic gradient. It is essential to clearly describe the technique employed here, particularly to interpret the values reported in Figure 2 and Supplementary Figure S1.

 2. **Readability and consistency of Supplementary Figure S1 and susceptibility data:**

 The presentation of Supplementary Figure S1 lacks clarity. It would be helpful to specify at which timepoints and for what reasons susceptibility testing was repeated on the seven isolates. Does the original table correspond to antibiograms performed at the date of each sampling? This should be explicitly stated.

 3. **Consistency in antibiotic nomenclature:**

 Standardisation of antibiotic labels is needed throughout the manuscript and figures. For example, *Ceftazidime* and *CAZ* appear interchangeably, which may cause confusion. Harmonising these terms consistently would improve the clarity of the data presentation.

 4. **Chronological presentation of susceptibility data:**

 To enhance the reader's understanding and better visualise the evolution of susceptibility profiles over time, it would be preferable to reorganise Table S1 into seven distinct tables (one per isolate), each showing the antibiogram results at the corresponding timepoint.

 5. **Justification for Average value calculations:**

 The mention of a calculated average in the susceptibility testing data lacks clinical or methodological justification in this context. It is not clear what the authors intend to demonstrate by calculating the mean for susceptibility testing diameter. The rationale behind using an average in this context is not evident, and it does not appear to provide meaningful insight into the data

 It is more meaningful to assess the trend or evolution of the diameters over time, as the mean value does not provide relevant or informative insight in this context

 6. **Improvement of the timeline figure:**

 In the timeline figure displaying sampling and treatment chronology, it would be helpful to include the calendar year along the time axis to contextualise the clinical events.

 7. **Clarification of figure symbols:**

 The meaning of the different symbols (circles, squares, triangles) used in the figure is not clearly explained. It would be useful to specify these in the legend or integrate this figure with the relevant table to improve readability.

 8. **Origin of isolate subcultures:**

 It is not indicated whether the subcultures were performed from fresh clinical samples or from previously frozen isolate stocks. This information is essential for interpreting phenotypic and genotypic evolution and should be specified.

 9. **Bacterial morphotypes and potential sub-populations:**

 It would be of interest to mention whether different bacterial colony morphotypes were observed on initial culture, as this could suggest the presence of sub-populations with differing resistance profiles.

 10. **Correlation between meropenem resistance phenotype and ARDaP prediction:**

 The correlation between the observed meropenem resistance phenotypes and ARDaP predictions is not entirely clear, particularly for isolate number 2 and the average. Clarification of this point in both the results and the figures would strengthen the manuscript.

Are enough details provided of any physical examination and diagnostic tests, treatment given and outcomes?

Partly

Is the case presented with sufficient detail to be useful for other practitioners?

Partly

Is sufficient discussion included of the importance of the findings and their relevance to future understanding of disease processes, diagnosis or treatment?

Partly

Is the background of the case’s history and progression described in sufficient detail?

Yes

Reviewer Expertise:

Melioidosis, antibiotic resistance mechanisms, with a particular focus on membrane-associated mechanisms such as efflux, as well as genomics and transcriptomics.

I confirm that I have read this submission and believe that I have an appropriate level of expertise to confirm that it is of an acceptable scientific standard, however I have significant reservations, as outlined above.
